# Bottom‐Up Synthesis of Metallic CoNi Nanoplatelets with Magnetic Vortex‐Like Spin Configurations

**DOI:** 10.1002/smsc.202500111

**Published:** 2025-04-23

**Authors:** Mena‐Alexander Kräenbring, Konstantin Bomm, Georg Bendt, Hanna Pazniak, Benjamin Zingsem, Thomas Feggeler, Sebastian Wintz, Simon Kempkens, Marina Spasova, Stephan Schulz, Michael Farle, Ulf Wiedwald

**Affiliations:** ^1^ Faculty of Physics and Center for Nanointegration Duisburg‐Essen University of Duisburg‐Essen 47057 Duisburg Germany; ^2^ Institute for Energy and Materials Processes – Particle Science and Technology (EMPI‐PST) University of Duisburg‐Essen Carl‐Benz‐Straße 199 47057 Duisburg Germany; ^3^ Faculty of Chemistry and Center for Nanointegration Duisburg‐Essen University of Duisburg‐Essen 45117 Essen Germany; ^4^ LMGP Grenoble‐INP Minatec 3 parvis Louis Néel CS 50257 38016 Cedex 1 Grenoble France; ^5^ Ernst Ruska‐Centre for Microscopy and Spectroscopy with Electrons Forschungszentrum Jülich 52425 Jülich Germany; ^6^ Advanced Light Source Lawrence Berkeley National Laboratory Berkeley 94720 CA USA; ^7^ National Synchrotron Light Source II Brookhaven National Laboratory Upton 11973 NY USA; ^8^ Institute for Nanospectroscopy Helmholtz‐Zentrum Berlin für Materialien und Energie 14109 Berlin Germany

**Keywords:** cancer theranostics, magnetic vortices, nanoplatelets, plasma reduction

## Abstract

Magnetic nanoplatelets hold significant potential for various technical applications due to their ability to switch between a fully magnetized state with high magnetization and a vortex‐like configuration that eliminates stray fields in the absence of an external field. This study presents the synthesis of uniform CoNi nanoplatelets through the topotactic reduction of metal hydroxides using hydrogen plasma. The reduction process is analyzed via magnetometry, leveraging the transition from paramagnetic hydroxide to ferromagnetic metal. Lorentz transmission electron microscopy and scanning transmission X‐ray microscopy confirm the presence of magnetic vortex‐like structures in isolated Co_0.85_Ni_0.15_ nanoplatelets at ambient temperature. Additionally, micromagnetic simulations are conducted to further explore the magnetic properties of the nanoplatelets, revealing the formation of magnetic vortex remanent states at diameters between 200 nm and 1 μm and a thickness of around 12 nm. Notably, structural defects and thickness variations do not directly destabilize the magnetic vortex configurations.

## Graphical abstract

The study presents the topotactic reduction of CoNi hydroxide nanoplatelets into metallic CoNi nanoplatelets with diameters of around 1 μm using a scalable bottom‐up approach. Their resulting high magnetization and magnetic vortex remanent state unlock exciting potential for advanced biomedical applications, including targeted diagnostics and therapy.

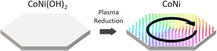

## 1 Introduction

Magnetic nanoparticles have emerged as powerful tools in biomedical applications due to their unique magnetic properties, biocompatibility, and ability to be manipulated by external magnetic fields.^[^ ^1^, ^2^, ^3^, ^4^, ^5^ ^]^ Their small size and high surface‐to‐volume ratio enable functionalization with various ligands, making them highly versatile for targeted drug delivery,^[^ ^6^ ^]^ magnetic resonance imaging (MRI),^[^ ^7^ ^]^ hyperthermia therapy,^[^ ^8^ ^]^ and biosensing.^[^ ^9^ ^]^ In particular, magnetic nanoparticles are widely explored for cancer theranostics, where they serve both diagnostic and therapeutic functions, offering a minimally invasive alternative to conventional treatments.^[^ ^10^, ^11^, ^12^ ^]^

Among the diverse types of magnetic nanostructures, magnetic nanoplatelets present distinct advantages. Due to their geometry, magnetic nanoplatelets can exhibit magnetic vortex remanent states, which have been extensively studied.^[^ ^13^, ^14^, ^15^, ^16^, ^17^, ^18^, ^19^, ^20^ ^]^ Magnetic vortex states enable the platelets to achieve a high saturation magnetization when magnetized and a negligible stray field in the absence of a magnetic field,^[^ ^16^, ^21^ ^]^ facilitating the formation of stable dispersions due to minimized magnetic interactions and thus mitigating one of the main sources of nanoparticle agglomeration.^[^ ^22^ ^]^ Upon applying an external magnetic field, a torque acts on magnetic nanodiscs with in‐plane magnetization, rotating them until their plane is parallel to the external field.^[^ ^23^ ^]^ In larger magnetic fields, the vortex structure dissolves and a large homogeneous in‐plane magnetization is obtained.^[^ ^14^ ^]^

Furthermore, lithographically produced permalloy nanoplatelets with magnetic vortex remanent states were employed to attack cancer cells in vitro, achieving 90% cell disintegration within 10 min.^[^ ^21^ ^]^ However, the usually employed top‐down approaches for the synthesis of nanoplatelets (such as expensive lithography) often suffer from poor scalability, whereas bottom‐up methods can parallelize and upscale the synthesis. Thus, the platelets can potentially be prepared more quickly, cheaply, and easily. While Fe_3_O_4_ platelets with magnetic vortex structures have been synthesized before using solvothermal techniques,^[^ ^24^ ^]^ other viable material compositions are still missing in literature. In this work, we aim to add one candidate system and report the topotactic reduction of CoNi hydroxide nanoplatelets, which are easily accessible via green and scalable homogeneous precipitation from aqueous solutions,^[^ ^25^, ^26^ ^]^ to metallic nanoplatelets with a diameter of about 1 μm, which are shown to possess magnetic vortex‐like remanent states. Homogeneous precipitation offers superior scalability and milder reaction conditions compared to solvothermal methods, which often require high pressures and specialized reactors. Given the dimensions of the platelets at different stoichiometries, the stoichiometry Co_0.85_Ni_0.15_ was the most promising for studying the magnetic vortex formation. In addition to experimentally determined parameters such as lateral size and thickness, the expected saturation magnetization and the ease of the reduction process were considered.

The larger magnetization of the metal nanoplatelets as compared to ferrimagnetic oxides could be advantageous for magnetomechanical excitations and enhanced remote steering in magnetic gradient fields.^[^ ^27^ ^]^ The biocompatibility of CoNi‐based materials was shown to be highly dose‐dependent, with doses as low as 6.25 μg mL^−1^ showing no undesirable side effects against healthy human cells while selectively inducing cell death in cancer cells.^[^ ^28^ ^]^ Furthermore, it is possible to enhance the biocompatibility of CoNi‐based materials via polymer coatings,^[^ ^29^ ^]^ rendering the platelets viable for biomedical applications.

## 2 Experimental Section

### 2.1 Synthesis

An amount of 6.5 mmol of cobalt nitrate hexahydrate (Co(NO_3_)_2_(H_2_O)_6_) and 4.0 mmol of hexamethylenetetramine (C_6_H_12_N_4_) were dissolved in 250 mL of deionized water, heated to 363 K (90 °C) and stirred for 1 h, yielding a pink suspension. After cooling the suspension to ambient temperature, the powder was isolated by centrifugation, washed thoroughly with deionized water and acetone, and dried. Co_0.85_Ni_0.15_(OH)_2_ nanoplatelets were analogously prepared using different mixtures of Co(NO_3_)_2_(H_2_O)_6_ and Ni(NO_3_)_2_(H_2_O)_6_. In contrast to the nanoplatelets synthesized via the method of Hu et al.^[^ ^30^ ^]^ and reduced via forming gas (which we discuss in Supporting Information), the nanoparticles obtained by this method did not contain any cubic nanoparticles on the platelets’ surfaces. They were then reduced by applying the reactive hydrogen plasma process described below.

### 2.2 Isolated Nanoplatelets on Substrates for Hydrogen Plasma Reduction

**Figure 1:**
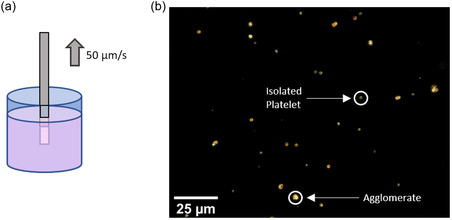
Dip‐coating geometry for a) platelet deposition and b) dark‐field optical microscopy image of the deposited platelets on Si/SiO_2_ substrate. Circles indicate an isolated platelet and a larger agglomerate which can be judged by the dark‐field contrast.

For structural and magnetic characterization, hydroxide nanoplatelets were first deposited on different substrates. For this purpose, 10 mg of Co_0.85_Ni_0.15_(OH)_2_ platelet powder was dispersed in 5 mL of ethanol in a sealable container. The container was closed and shaken for a minute to distribute the platelets evenly in the ethanol. While large agglomerates sediment fast, single nanoplatelets and smaller clusters of platelets sedimented slow enough to transfer them onto Si/SiO_2_ substrates and transmission electron microcopy (TEM) grids using a dip coater. The substrate was partially immersed in the dispersion and then slowly pulled out at a constant velocity of 50 μm s^−1^ using a custom‐made dip coater as shown in **Figure** **1**a. At this velocity, we reproducibly obtained platelet densities on the substrates resulting in a total magnetic moment large enough to be measured by vibrating sample magnetometry (VSM). At the same time, the density was small enough to yield a significant number of isolated platelets at distances much larger than their lateral dimensions. Thus, magnetic dipole–dipole interactions could be neglected for such isolated platelets, which was critical for the characterization of the magnetic state when imaging spin configurations. Figure 1b shows a dark‐field optical microscopy image of Co_0.85_Ni_0.15_(OH)_2_ platelets on Si/SiO_2_. Single platelets could be identified at low contrast while agglomerates appeared brighter as confirmed by scanning electron microscopy (SEM). Lower dip‐coating velocities resulted in a more inhomogeneous but overall higher platelet density on the substrates while higher velocities led to more homogeneous but overall lower platelet densities.

The samples were then exposed to an inductively coupled hydrogen plasma operating at 13.56 MHz to transform the platelets to the metallic state. Comparable process conditions were previously successfully applied for the reduction of nanoparticles and thin films and it was known that the reduction of Co and Ni was most efficient at elevated temperatures of up to 673 K (400 °C). At this temperature, surfactants were also effectively removed from the formed metallic surfaces.^[^ ^31^, ^32^, ^33^, ^34^, ^35^ ^]^ For the CoNi hydroxide platelets on Si/SiO_2_, the plasma was set to a power of 50 W at a hydrogen pressure of 13 Pa. The substrate temperature during reduction was varied between ambient temperature and 673 K (400 °C) and measured by a pyrometer. The duration was varied between 20 and 40 min. The optimal parameters were 40 min at 570 K (297 °C) as judged by SEM and VSM.

### 2.3 Structure and Morphology

The size, morphology, and distribution of platelets on the substrates were examined by a Zeiss Leo 1530 SEM with an acceleration voltage of 20 kV using the in‐lens detector and a Zeiss Axiotech optical microscope. A JEOL JSM‐6510 SEM with Bruker Quantax 400 EDX was used for the characterization directly after synthesis. X‐Ray powder diffraction (XRD) patterns of hexagonal Co_x_Ni_1−x_(OH)_2_ platelets were recorded at ambient temperature using a Bruker D8 Advance powder diffractometer in Bragg–Brentano geometry using Cu‐K_α_ radiation (λ = 0.15418 nm). The powder samples were investigated in the 2*θ* range of 5°–90° with a step size of 0.01° and a counting time of 0.3 s per point.

The crystal structure and morphology of the metallic nanoplatelets were investigated using a C_S_‐corrected JEOL 2200FS transmission electron microscopy (TEM) device at an acceleration voltage of 200 kV. For this purpose, the platelets were transferred onto an Formvar‐covered (Vinylec) TEM grid (Plano, Germany) by manually dipping the TEM grid into the colloidal suspension containing the platelets for about 3 s. The particles were then reduced at 570 K (297 °C) over a period of 40 min using a hydrogen plasma, followed by an in situ carbon coating (roughly 20 nm thick) to protect the platelets from oxidation. Si/SiO_2_ substrates were used for magnetometry and Si_3_N_4_ membranes for scanning transmission electron microscopy (STXM) measurements.

### 2.4 Magnetic Properties

The magnetic properties of the nanoplatelets were determined by VSM using a Quantum Design DynaCool PPMS system in ±9 T and variable temperature (5–300 K). Lorentz microscopy was conducted in a Jeol 2200FS TEM (see previous sections). Room‐temperature, spatially resolved X‐Ray magnetic circular dichroism (XMCD) measurements were performed at the MAXYMUS scanning transmission X‐ray microscope at the BESSY II synchrotron facility at the Helmholtz Center Berlin. The sample was mounted at an angle of 30° toward the X‐ray incidence and scanned using an 18 nm zone plate and a dwell time of 5 ms per pixel. The X‐ray energy was set to the Co‐L_3_ absorption edge maximum at nominally 779.5 eV as obtained from the normalized X‐ray absorption spectrum shown in Figure 7a.

### 2.5 Micromagnetic Simulations

Micromagnetic simulations for Co_0.85_Ni_0.15_ platelets of various sizes (200–1000 nm) were conducted using mumax^3^ with a GTX1650‐Super graphics card by Nvidia, employing driver version 536.23 and CUDA V11.3.109.^[^ ^36^, ^37^ ^]^ A discretization cell size of 2×2×2 nm^3^ was chosen which is less than half the calculated exchange length of about 5 nm. To determine the magnetic state, the voxel magnetic moments were assigned to a random direction in the beginning. Then, the system was relaxed to its minimum energy. The exchange constant (2.7 × 10^−11^ J · m^−1^) and the saturation magnetization (1.26 × 10^6^ A · m^−1^) for Co_0.85_Ni_0.15_ were linearly interpolated from literature values of pure cobalt and nickel.^[^ ^38^, ^39^ ^]^ Such approximation was based on the Slater–Pauling curve, which shows a linear progression of the saturation magnetization.^[^ ^40^, ^41^ ^]^ A thickness of 12 nm was chosen for all simulated platelets. All other parameters were left to their default values.

## 3 Results and Discussion

### 3.1 Micromagnetic Simulations

Given the dimensions of the platelets at different stoichiometries, the stoichiometry Co_0.85_Ni_0.15_ was the most promising for studying the magnetic vortex formation. The magnetic configuration of Co_0.85_Ni_0.15_ hexagonal platelets (200–1000 nm, thickness 12 nm) was determined by micromagnetic simulations using mumax^3^.^[^ ^36^ ^]^ The simulation parameters are given in Experimental Section. For each magnetic element, the magnetization direction was set random as a starting point. Then, the system was energetically relaxed in a zero‐field environment until an energetic minimum was found. For these simulations, the magneto‐crystalline anisotropy has been neglected.

**Figure 2:**
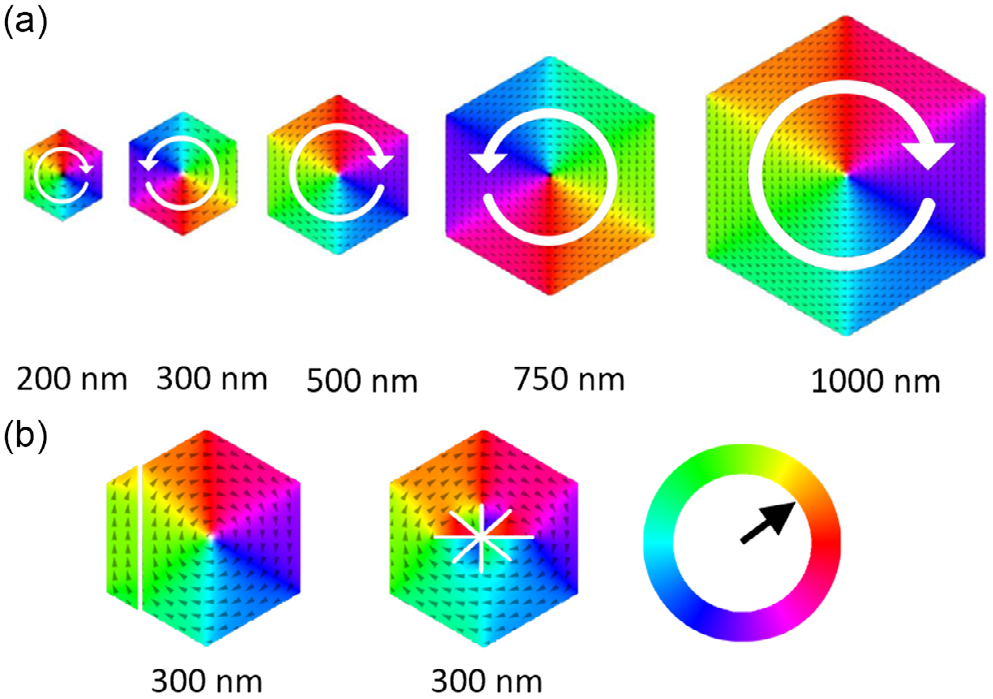
a) Micromagnetic simulations of defect‐free Co_0.85_Ni_0.15_ platelets. The corner‐to‐corner distance is indicated. The thickness is set to 12 nm for all platelets. b) Micromagnetic simulations of Co_0.85_Ni_0.15_ platelets with defects obtained by TEM as indicated by the white lines. The color wheel indicates the direction of the simulated cells’ magnetic moments.

The magnetic configurations are shown in **Figure** **2**a. The corner‐to‐corner distance on opposite sides (diameter) was varied between 200 and 1000 nm in the micromagnetic simulations, revealing that the remanent magnetic configuration is a vortex state in all cases. This aligns with similar simulations on magnetite‐based platelets,^[^ ^24^ ^]^ emphasizing the critical role of saturation magnetization in stabilizing vortex states. We further investigated possible deviations from the ideal platelet structure as shown in Figure 2b. The most common defects, i.e., broken edges or central gaps, were simulated. The overall vortex state was not significantly affected by such defects. Dipole–dipole interactions with neighboring platelets were not considered.

### 3.2 Hydroxide to Metallic Nanoplatelet Reduction by Hydrogen Plasma

**Figure 3:**
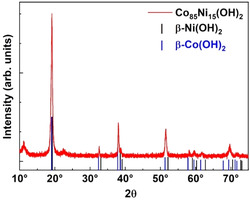
XRD data of Co_0.85_Ni_0.15_(OH)_2_ nanoplatelets using copper K_α_ radiation (λ = 0.15418 nm). The sticks represent the positions and intensities of the *β*‐Co(OH)_2_ (COD:9 009 101) and *β*‐Ni(OH)_2_ (COD:9 009 112) powder references.

We obtained *β*‐Co(OH)_2_ and *β*‐Ni(OH)_2_ hexagonal nanoplatelets and the full compositional range of Co_x_Ni_1‐x_ with 0 ≤ × ≤ 1 has been synthesized as described and shown in the text and Figure S1–S6, Supporting Information. **Figure** **3** presents XRD data of Co_0.85_Ni_0.15_(OH)_2_ platelets, revealing the formation of the *β*‐phase. The reflexes appear in between those of the pure *β*‐Co(OH)_2_ and *β*‐Ni(OH)_2_ powder reference data but somewhat closer to the *β*‐Co(OH)_2_ as expected.

**Figure 4:**
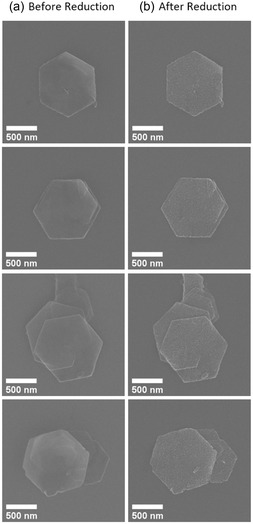
SEM images of the same platelets a) before and b) after reduction in hydrogen plasma at 570 K (297 °C) for 40 min. While the platelet morphology is maintained, a slight increase in the surface roughness is discernible.

Co_0.85_Ni_0.15_(OH)_2_ nanoplatelets were reduced to the metallic state on Si/SiO_2_ substrates, TEM grids, and Si_3_N_4_ membranes as supports using a hydrogen plasma process as described in Experimental Section. A selection of SEM images of single nanoplatelets and small agglomerates before and after the optimized hydrogen plasma reduction at 570 K (297 °C) for 40 min is shown in **Figure** **4**. The overall hexagonal shape of the platelets is maintained while the surface roughness appears to have increased. Interestingly, a corkscrew‐like growth mode is identified connecting the platelets in some small agglomerates. In these experiments, the platelets were not covered by a protective carbon layer. Furthermore, only very few defects such as gap formations or broken edges were found. STEM‐EDX measurements (Figure S7, Supporting Information) confirmed a homogeneous distribution of Co and Ni within a single platelet, with the targeted Co_0.85_Ni_0.15_ stoichiometry achieved within the technique's error margin.

Samples on TEM grids were in situ covered with carbon to avoid oxidation and maintain the metallic state. The size distribution of the Co_0.85_Ni_0.15_ platelet batch is shown in Figure S8, Supporting Information. Hexagonal platelets, measured across opposite corners, range in size from 200 to 1400 nm, with the most probable sizes between 800 and 900 nm. In this size range, the formation of magnetic vortex structure is expected as shown earlier by micromagnetic simulations. Additionally, the contribution of the magneto‐crystalline anisotropy to the global energy landscape of a platelet makes the formation of an at least vortex‐like noncollinear spin structure likely.

Figure S9, Supporting Information, shows a selected area electron diffraction pattern of a single‐Co_85_Ni_15_ nanoplatelet. The reciprocal lattice parameters were measured and compared to the calculated lattice parameters of hexagonal Co_0.85_Ni_0.15_^[^ ^42^ ^]^ in Table S1, Supporting Information. The results are in excellent agreement with the literature. The observation of various spots at different reciprocal lattice distances and the ringlike smearing of the individual reflexes are indicative of a polycrystalline lattice textured by highly preferential directions. The determined crystal structure is in agreement with the crystal phase diagram of Co and Ni alloys,^[^ ^42^ ^]^ even though defective Co_0.85_Ni_0.15_ nanoplatelets are also observed (Figure S10, Supporting Information). During reduction, the shown platelet on the TEM grid fragments into numerous irregularly shaped nanoparticles while largely retaining its initial hexagonal form. This behavior is attributed to stress experienced during the reduction process. Thus, when platelets adhere strongly to the support, structural integrity is compromised, leading to their disintegration into irregular metallic nanoparticles.

### 3.3 Magnetic Characterization of Metallic Co_0.85_Ni_0.15_ Platelets

**Figure 5:**
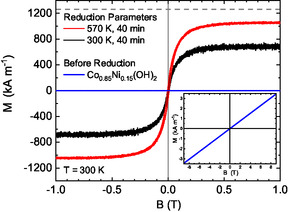
VSM measurements of platelets on Si substrates at 300 K in in‐plane geometry reduced at 570 K (red) and 300 K (black) for 40 min and the paramagnetic Co_0.85_Ni_0.15_(OH)_2_ hydroxide powder (blue). The dashed line shows the saturation magnetization of volumetric Co_0.85_Ni_0.15_ (M = 1260 kA m^−1^). The saturation magnetization of the platelets reduced at 570 K reaches 81% of bulk magnetization while the error bar is estimated to be about 40% due to the uncertainty of the total volume. The inset presents the paramagnetic response of the hydroxide powder at 300 K.

The magnetic properties were determined by VSM, Lorentz TEM, and STXM. Integral VSM measurements were used to judge the success of the reduction process. The samples were first reduced at different temperatures ranging from 300 to 670 K and then covered with a nominally 20 nm thick layer of carbon to prevent the reoxidization of the nanoplatelets during ex situ transfer. Investigations by SEM revealed that reduction temperatures of 630 K and above damaged the platelets. **Figure** **5** shows the field‐dependent magnetization of the Co_0.85_Ni_0.15_(OH)_2_ platelet powder (blue) and two metallic platelet samples reduced at 300 K (black) and 570 K (red) for 40 min in situ covered by carbon.

After the hydrogen plasma reduction, the saturation magnetization increased drastically. At 300 K, the measured saturation magnetization reaches about half the volumetric value of M_S_ = 1260 kA m^−1^. This might be due to an incomplete reduction of small agglomerates present on the Si/SiO_2_ substrates. The optimal reduction temperature was found to be 570 K since the platelets remained largely undamaged and the saturation magnetization M_S_ = 1050 kA m^−1^ reached the highest value of 81% of the volumetric M_S_ of Co_0.85_Ni_0.15_. Note that it is difficult to determine the total sample volume for normalization. We estimate the error bar to be up to 40%. Thus, the measured magnetization reflects the bulk magnetization and the results clearly show that most of the platelets have been reduced to the metallic state and are thus ferromagnetic. The coercive field of μ_0_H = 12 mT was rather low and the remanent magnetization was only about 100 kA m^−1^, which is less than 10% of M_S_ in in‐plane geometry. These findings agree with the expectations for magnetic vortices: a low coercive field and a near‐zero remanent magnetization. Compared to magnetite, (M_S_ = 490 kA m^−1^)^[^ ^24^ ^]^ Co_0.85_Ni_0.15_ (M_S_ = 1260 kA m^−1^) exhibits a 2.5 times larger volume magnetization and is thus more attractive for remote magnetic steering and magnetomechanical actuation.

**Figure 6:**
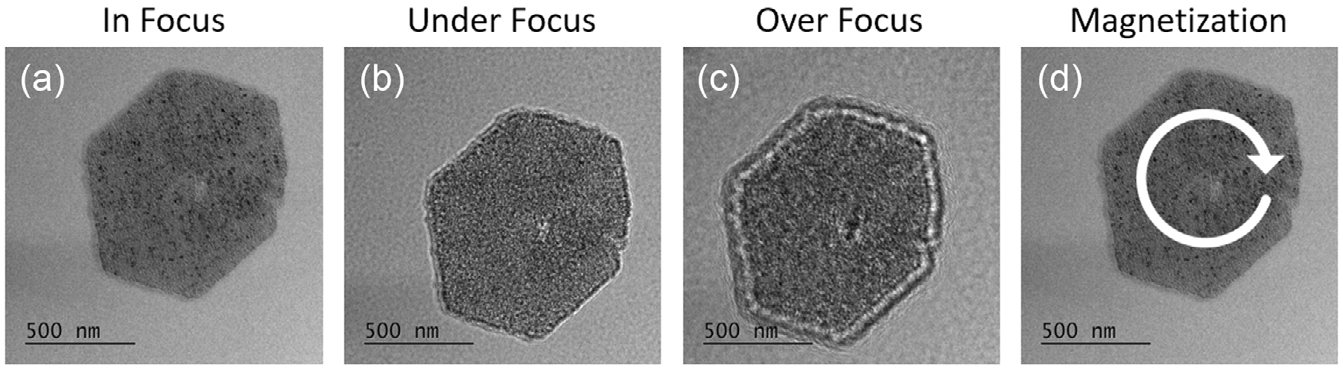
a) The in‐focus, b) under‐focus, c) over‐focus Lorentz‐TEM measurements, and d) the resulting remanent magnetization state, respectively.

Single‐ferromagnetic platelets were further investigated by Lorentz TEM to shed light on the spin configuration. For Lorentz TEM, the platelets were transferred onto a TEM grid by dipping the TEM grid into the platelet dispersion for a few seconds. The platelets were then reduced using the optimal parameters and covered with amorphous carbon. About 10% of the platelets on the TEM grid were found sufficiently isolated. The majority is concentrated in small agglomerates which are not considered in the following due to dipolar interactions. Nonetheless, hundreds of isolated platelets were present and investigated by Lorentz TEM. In such magnetic field–free conditions, about 5% of the isolated platelets exhibited a bright or dark spot in the center with contrast inversion under focus inversion, pointing to the presence of a magnetic vortex‐like remanent state, an example is shown in **Figure** **6**. However, the faded bright spot at the center of the platelet in the in‐focus image may indicate that the observed signal originates from topographic thinning or structural defects instead of remanent magnetic configuration. The video file in the Supporting Information shows the contrast inversion while the focus is adjusted. The yield of isolated platelets, and by extension magnetic vortices, could be further improved by exploring different nanoplatelet dispersion media, solid concentration ranges, and surfactants, as well as by incorporating additional low‐shear dispersion and layer deposition techniques as well as fine‐tuned plasma reduction conditions. Given that all platelets on the grid were exposed to the same magnetic fields during bright field imaging and alignment before Lorentz imaging, the presence of both, dark and bright contrast, in the initial state indicates that no chiral magnetic interaction is present to define a preferred vortex orientation.

**Figure 7:**
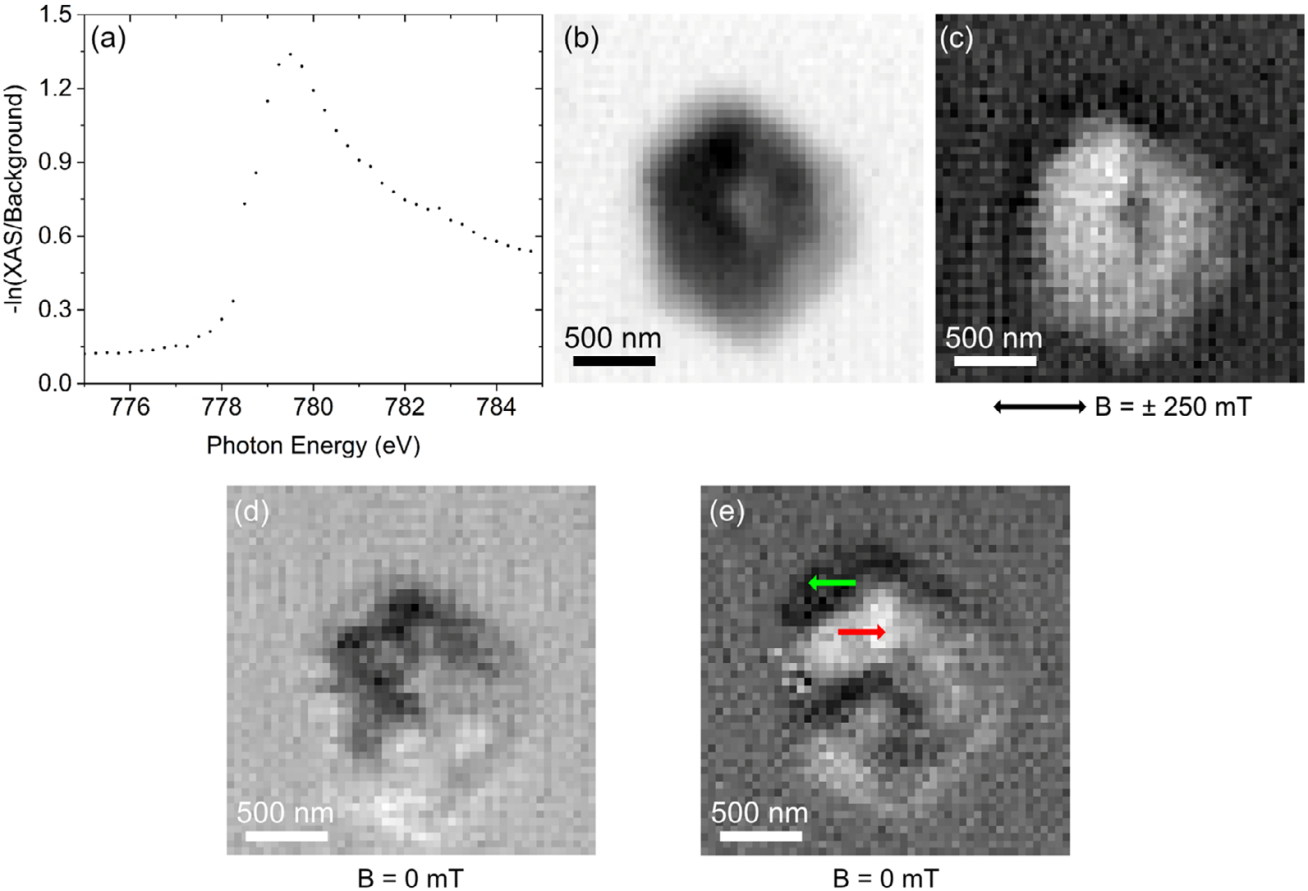
a) Normalized X‐Ray absorption spectrum of the Co L_3_ edge, b) XAS image, and c) XMCD image (+B/‐B) recorded at ± 250 mT/σ^−^ X‐Ray polarization. d,e) XMCD images (σ^+^/σ^−^) at 0 mT. The field direction is indicated by the black arrow, the magnetization orientation by colored arrows in panel (e).

The spin configuration in isolated platelets was further investigated via STXM to prove vortex states in the nanoplatelets. The chemical state of the reduced platelets can be easily judged from X‐Ray absorption spectroscopy (XAS), and the element‐specific spin configuration is obtained from imaging at the maximum XMCD signal at the respective absorption edge. We have chosen the Co‐L_3_ edge due to the high amount of Co in the Co_0.85_Ni_0.15_ platelets. **Figure** **7**a shows the XAS of the Co‐L_3_ edge. The line shape reflects the typical spectrum expected for metallic Co, and the absence of sharp features expected for Co^2+^ in oxidic environments proves the complete reduction of the platelets and highlights their long‐term stability after covering them with amorphous carbon.^[^ ^33^ ^]^ The spin configuration was investigated in several platelets, and two examples are presented. Figure 7b,c shows images of a single Co_0.85_Ni_0.15_ platelet obtained with σ^−^ polarization of X‐rays (Figure 7b) with absorption contrast and (Figure 7c) with normalized magnetic contrast (images at ± 250 mT in‐plane field were divided by each other). A rather single‐domain state of the magnetization is observed at ± 250 mT, indicated by the widely homogeneous light gray XMCD contrast with a small region at the center showing darker contrast, that can also be seen as a topographic defect/hole in the absorption contrast. Compared to the integral VSM hysteresis loop (Figure 5), we reach about 90% of the saturation value confirming a single‐domain state in the field. Removal of the external magnetic field results in the formation of a noncollinear spin configuration.

Figure 7d shows a vortex‐like configuration, indicating typical characteristics of such a magnetic configuration in its infancy. Here, images recorded with both σ^+^ and σ^−^ have been divided to gain normalized magnetic contrast. Despite not perfectly aligned, the XMCD contrast changes from black between the 10 and 12 o'clock positions, counterclockwise to dark grey at the 9 o'clock position, toward light grey/white at the 6 o'clock position, to successively return to the initial black. In contrast, the example platelet shown in Figure 7e (magnetization orientation is indicated by colored arrows) exhibits rather a swirl‐like domain structure, suggesting the onset of a noncollinear circular spin configuration.

## 4 Conclusions

Hexagonal Co_0.85_Ni_0.15_(OH)_2_ nanoplatelets were synthesized using a green and scalable homogeneous precipitation technique.^[^ ^25^, ^26^ ^]^ In this context, dip coating has proven to be a reliable method to isolate individual nanoplatelets on a substrate. Hydrogen plasma treatment reliably reduced the isolated hydroxide platelets to the metallic state while maintaining their overall shape. The resulting Co_0.85_Ni_0.15_ nanoplatelets exhibit a bulk‐like magnetization and allowed the stabilization of magnetic vortex‐like remanent states as imaged by Lorentz TEM and STXM. Micromagnetic simulations revealed that the remanent state for platelets of this size range is a vortex state and that structural defects in the platelets do not directly lead to its destabilization.

## Conflict of Interest

The authors declare no conflict of interest.

## Author Contributions

**Mena‐Alexander Kräenbring**: data curation (lead); formal analysis (lead); investigation (lead); methodology (equal); validation (equal); visualization (equal); writing—original draft (lead). **Konstantin Bomm**: investigation (equal); methodology (supporting); validation (supporting). **Georg Bendt**: conceptualization (equal); methodology (equal); project administration (equal); resources (lead); supervision (equal); writing—review editing (equal). **Hanna Pazniak**: investigation (equal); methodology (equal). **Benjamin Zingsem**: data curation (equal); formal analysis (equal); investigation (equal); methodology (supporting); validation (equal); visualization (equal). **Thomas Feggeler**: formal analysis (equal); investigation (equal); methodology (equal); visualization (supporting); writing—original draft (equal). **Sebastian Wintz**: data curation (supporting); formal analysis (equal); investigation (equal); methodology (supporting); validation (equal); visualization (equal); writing—review editing (equal). **Simon Kempkens**: data curation (supporting); formal analysis (supporting); investigation (equal); methodology (supporting). **Marina Spasova**: conceptualization (equal); investigation (supporting); methodology (supporting). **Stephan Schulz**: conceptualization (equal); funding acquisition (equal); project administration (equal); resources (equal); supervision (equal); writing—review editing (supporting). **Michael Farle**: conceptualization (equal); funding acquisition (equal); project administration (equal); resources (equal); writing—review editing (equal). **Ulf Wiedwald**: conceptualization (lead); data curation (equal); formal analysis (equal); funding acquisition (equal); investigation (equal); methodology (equal); project administration (lead); resources (equal); supervision (lead); validation (equal); visualization (supporting); writing—original draft (lead); writing—review editing (lead).

## Supporting information

Supplementary Material

## Data Availability

The data that support the findings of this study are available from the corresponding author upon reasonable request.
