# Supplementary material for: Bottom‐Up Synthesis of Metallic CoNi Nanoplatelets with Magnetic Vortex‐Like Spin Configurations
Source: Small Sci. 2025 Apr 23;5(7):2500111. doi: 10.1002/smsc.202500111 (PMC12257897; doi:10.1002/smsc.202500111)
Supplement: Supplementary file 1 — Supplementary Material [file SMSC-5-2500111-s001.zip › Smsc.202500111-sup-0001-suppdata-S1/Supporting Information Vortex Formation Platelets_Revision.pdf]

Supporting Information on

Bottom-up Synthesis of Metallic CoNi Nanoplatelets with Magnetic Vortex-Like Spin Configurations

Mena-Alexander Kräenbring[1,#], Konstantin Bomm[1], Georg Bendt[2], Hanna Pazniak[1,&], Benjamin Zingsem[1,3], Thomas Feggeler[4,⊥], Sebastian Wintz[5], Simon Kempkens[2], Marina Spasova[1], Stephan Schulz[2], Michael Farle[1], and Ulf Wiedwald[1]*

[1] Faculty of Physics and Center for Nanointegration Duisburg-Essen, University of Duisburg-Essen, 47057 Duisburg, Germany

[2] Faculty of Chemistry and Center for Nanointegration Duisburg-Essen, University of Duisburg-Essen, 45117 Essen, Germany

[3] Ernst Ruska-Centre for Microscopy and Spectroscopy with Electrons, Forschungszentrum Jülich, 52425 Jülich, Germany

[4] Advanced Light Source, Lawrence Berkeley National Laboratory, Berkeley CA 94720, United States

[5] Helmholtz-Zentrum Berlin für Materialien und Energie, 14109 Berlin, Germany

[#]new address: Institute for Energy and Materials Processes – Particle Science and Technology (EMPI-PST), University of Duisburg-Essen, Carl-Benz-Straße 199, 47057 Duisburg, Germany

[&]new address: LMGP Grenoble-INP, Minatec 3, parvis Louis Néel CS 50257 38016 Grenoble Cedex 1, France

[⊥]new address: National Synchrotron Light Source II, Brookhaven National Laboratory, Upton NY 11973, United States

[*]corresponding author E-mail: ulf.wiedwald@uni-due.de

1. Alternative Synthesis of $Co_xNi_{1-x}(OH)_2$ platelets via the method of Hu et al.

In an alternative synthesis, hexagonal $Co_xNi_{1-x}(OH)_2$ platelets were synthesized using $Co(NO_3)_2$ and $Ni(NO_3)_2$ precursors according to the protocol of Hu *et al.*[1] Different stoichiometries were achieved by variation of the molar ratio of the metal salts. For example, $Co_{80}Ni_{20}$ hydroxides nanoplatelets were synthesized using a solution of 0.8 mmol $Co(NO_3)_2$ and 0.2 mmol $Ni(NO_3)_2$ in 20 mL of deionized water, to which a solution of 10 mL of ethanol and 2 mL of oleylamine was dropwise added over a period of 30 minutes. The resulting solution was subsequently heated to 453 K (180 °C) for 20 hours in an autoclave, yielding a crystalline powder that was isolated by centrifugation. After washing this powder twice with ethanol and once with acetone, the resulting material was dried in vacuo and ground to a fine powder using mortar and pestle. Nanoplatelets synthesized according to this route, which typically contained small cubic nanoparticles on the platelets' surfaces (Figure S3), were then used for reduction experiments using forming gas.

2. Calcination in Air and Forming Gas Synthesis of Metallic $Co_{1-x}Ni_x$ Nanoplatelets

The synthesis of $Co_{1-x}Ni_x$ hydroxide nanoplatelets was successfully conducted in composition steps of 25 at.% using the method described by Hu *et al.*[1] Materials formed as shown by X-ray diffraction (XRD, Figure S1) of powder samples demonstrating the formation of crystalline β-$Co(OH)_2$ and β-$Ni(OH)_2$ phases, which both crystallized in the $P\overline{3}m1$ space group with lattice parameters of a = 318.2 pm, c = 465.8 pm and a = 312.7 pm, c = 460.0 pm, respectively, which is in very good agreement with earlier results.[2,3] All compositions ranging from pure Co to pure Ni hydroxides showed identical phases and the lattice parameter gradually changed as indicated by the dashed line in Figure S1. Although hexagonal nanoplatelets were obtained for all compositions as shown in the scanning electron microscopy (SEM) images in Figure S2, each synthesis leads to individual size distributions. The mean corner-to-corner distances in the nanoplatelets were found to vary from about 250 nm to 400 nm.

The metal hydroxide platelets were then calcined in air at 573 K (300 °C) for 60 min, resulting in the formation of oxide materials as is exemplarily shown for Co (Figure S3). Subsequently, the resulting Co_3O_4 nanoplatelet powder was reduced in a mixture of argon and ethanol in a tube furnace at 573 K (300 °C) for 3h. The final temperature was reached within a few minutes. It is expected that Co_3O_4 is reduced to CoO. Further, the platelets were topotactically reduced to the metallic state upon heating to 573 K (300 °C) in an atmosphere of forming gas (95% Ar and 5% H_2) for 2 hours. The resulting metallic platelets are pyrophoric and are immediately oxidized upon exposure to air. The XRD data in Figure S4(a) exemplarily presents diffractograms for pure Co platelets after synthesis and after the reduction process in formation gas. The Co platelets crystallize in the hcp phase as indicated by the respective peak positions of powder reference diffractograms in Figure S4(a).

However, in further investigations, we focused on $Co_{0.8}Ni_{0.2}$ platelets for the forming gas reduction. Figure S4(b) presents SEM images before and after reduction showing that the hexagonal shape could not be fully maintained. The EDX analysis of $Co_{0.8}Ni_{0.2}$ platelets in Figure S4(c) reveals the composition of $Co_{0.80\pm0.02}Ni_{0.20\pm0.02}$. Figure S4(d) shows a TEM image of an isolated, highly symmetric hexagonal $Co_{0.8}Ni_{0.2}$ platelet.

We further characterized the magnetic properties before and after the reduction step. The magnetic response of a $Co_{0.8}Ni_{0.2}(OH)_2$ powder sample is shown in Figure S4(e). The sample is paramagnetic at T = 300 K while an open hysteresis and a strong paramagnetic slope are obtained at T = 5 K (see inset). β-$Co(OH)_2$ and β-$Ni(OH)_2$ are both antiferromagnets with Néel temperatures of 11.6 K and 25.8 K, respectively,[4,5] explaining the increased slope at T = 5 K. We assigned the open loop to uncompensated magnetic moments at the particle surface. Figure S5 presents the temperature dependence of the magnetization in a ZFC/FC protocol at B = 10 mT. Two peaks were obtained at T_{P1} = 7.2 K and T_{P2} = 31.2 K. The first peak is in good agreement with the work of Wang and Seehra where such a peak in the ZFC/FC curves is found at T = 9.5 K for β-$Co(OH)_2$,[4] while the second peak originates from Co_3O_4 formed due to air exposure before magnetometry. The onset of magnetic order (Néel temperature) is found by the deviation of a paramagnetic response by χT plots as shown in Figure S5(b). T_{N1} = 11.9 K and T_{N2} = 40.7 K are in very good agreement with the volumetric β-$Co(OH)_2$ Néel temperature of 11.6 K and the Co_3O_4 T_N = 40 K.[6]

Figure S4(f) presents hysteresis loops of metallic platelets at T = 5 K and T = 300 K. The saturation magnetization of M_S = 36 $Am^2 \cdot kg^{-1}$ at T = 300 K reflects the success of the reduction process since the magnetization rises by more than one order of magnitude as compared to the hydroxide and the expected ferromagnetic response at low and ambient temperature is obtained. The reduced M_S as compared to the estimated volumetric value of about M_S = 142 $Am^2 \cdot kg^{-1}$ obtained by linear interpolation between the nickel and cobalt saturation bulk magnetizations[7] results from significant oxidation of the platelet powder before entering the magnetometer. We suggest an alternative processing in the main text allowing us to characterize the pure metallic state.

The synthesis route suggested by Hu *et al.*[1] and the subsequent forming gas reduction is thus suitable for producing $Co_{1-x}Ni_x$ platelets in the complete stoichiometry range $0 \leq x \leq 1$. However, small amounts of cubic nanoparticles on the platelet surface are a typical byproduct of the synthesis (cf. Figure S3). In contrast, the alternative synthesis in the main text (cf. Methods section) avoided the formation of inhomogeneous nanoparticles and yielded high-quality platelets.

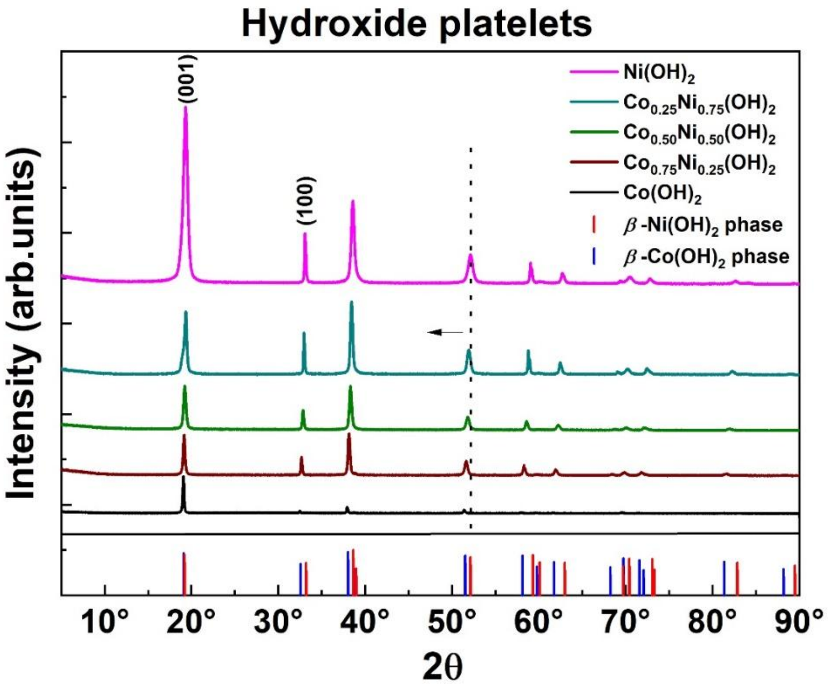

Figure S1: Set of XRD diffractograms with compositions between β-Co(OH)$_2$ and β-Ni(OH)$_2$ with 25% composition steps before subsequent processing steps. The black dashed line emphasizes the gradual shift from β -Co(OH)$_2$ to β-Ni(OH)$_2$ keeping the structure.

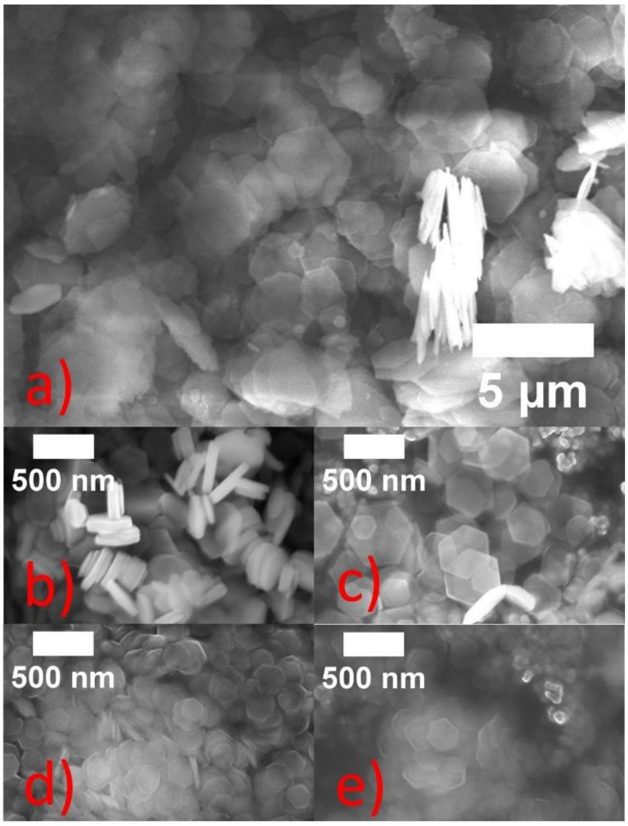

Figure S2: SEM images of hexagonal hydroxide platelets with Co/Ni compositions of Co (a), $Co_{0.75}Ni_{0.25}$ (b), $Co_{0.5}Ni_{0.5}$ (c), $Co_{0.25}Ni_{0.75}$ (d), and Ni (e).

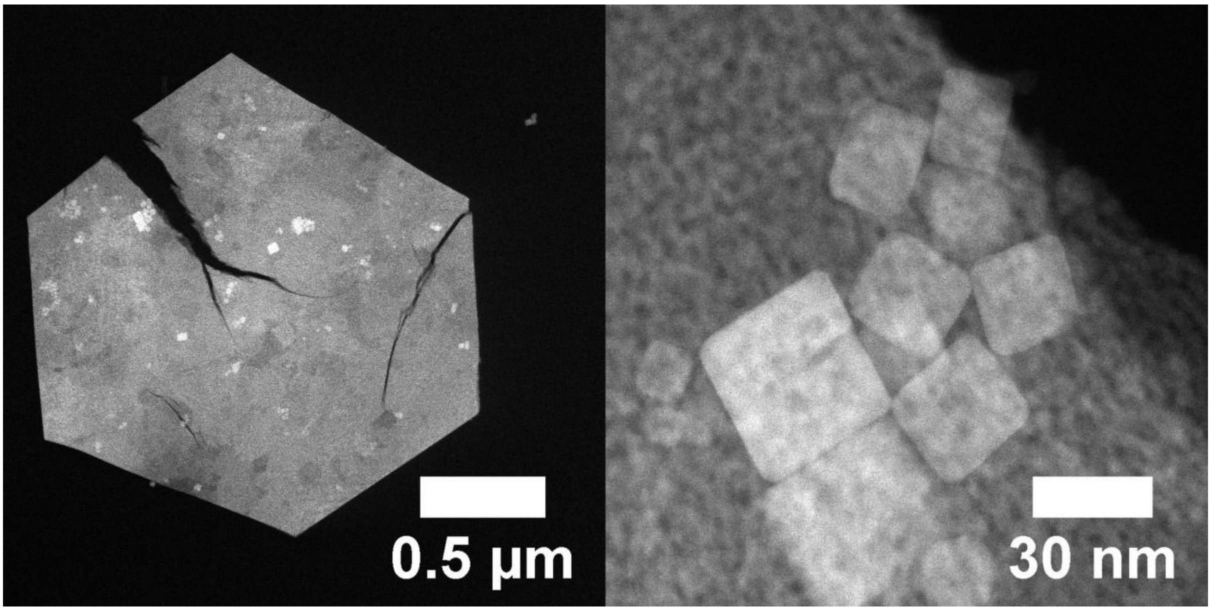

Figure S3: Scanning Transmission Electron Microscopy (STEM) of a Co_3O_4 platelet (left). In a higher magnified image (right) Co_3O_4 cubes are identified on the surface of the platelet which are by-products of the synthesis.

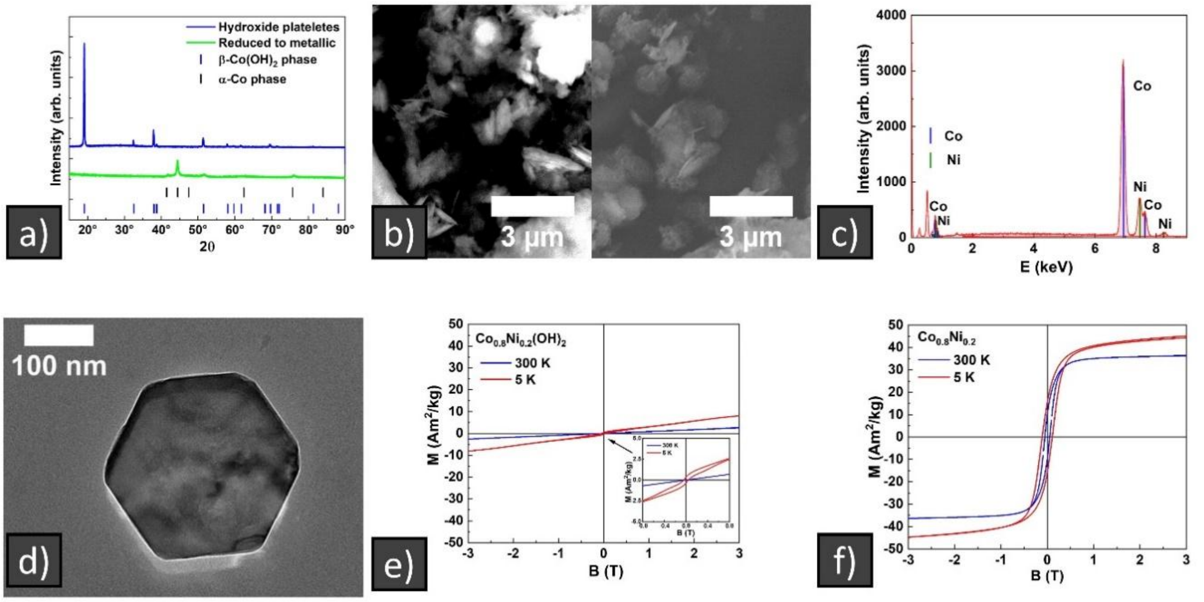

Figure S4. Structural and morphological characterization of hydroxide and metallic nanoplatelets. (a) XRD measurements of the cobalt hydroxide before and after reduction in forming gas. (b) SEM images of $Co_{0.8}Ni_{0.2}$ platelets before (left) and after (right) the reduction in forming gas. (c) EDX measurement after the reduction process for $Co_{0.8}Ni_{0.2}$ platelets. (d) TEM image of an isolated hydroxide platelet after synthesis. (e) and (f) VSM measurement of the platelets before and after the reduction process at T = 5 K and T = 300 K.

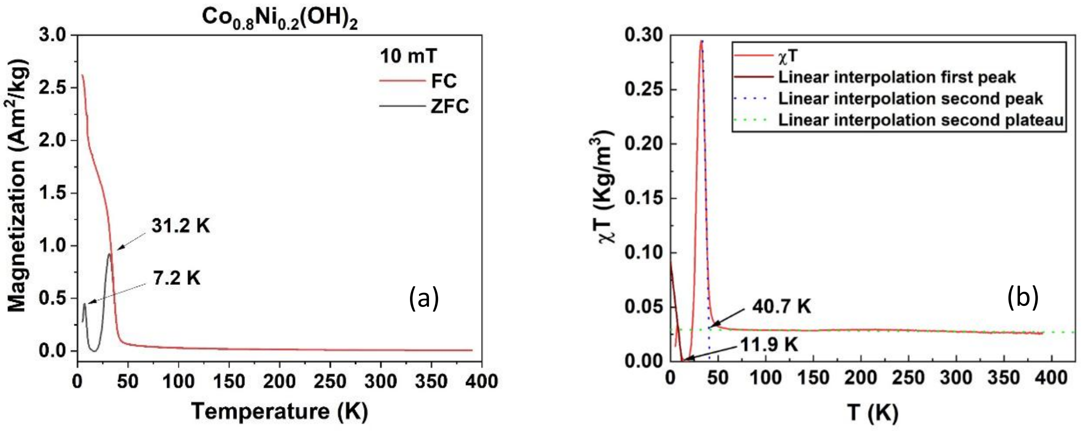

Figure S5: (a) ZFC/FC curves in B = 10 mT of $Co_{0.8}Ni_{0.2}(OH)_2$ indicating the antiferromagnetic order in two different phases, β-$Co(OH)_2$ and the byproduct Co_3O_4. (b) χT plot of the FC data in (a) for the determination of ordering temperatures. The constant values above 50 K indicate paramagnetism; below 50 K, the ordering temperatures of β-$Co(OH)_2$ T_{N1} = 11.9 K and Co_3O_4 T_{N2} = 40.7 K are identified.

3. Synthesis via the contamination-free homogeneous precipitation method

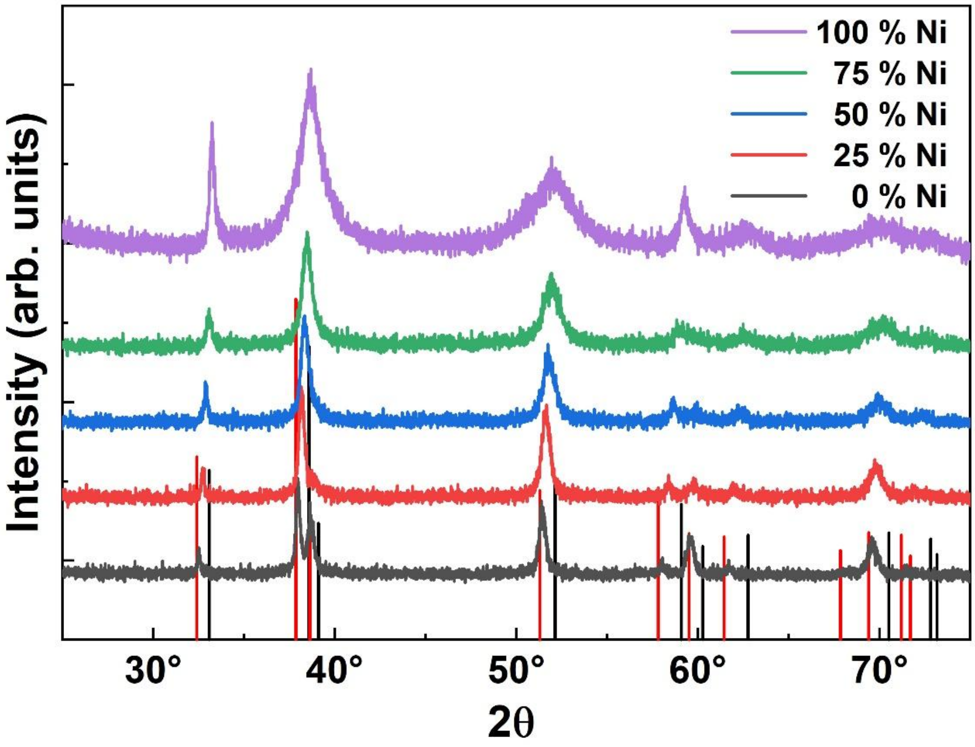

Figure S6. XRD-Data of $Co_{1-x}Ni_x(OH)_2$ synthesized following the second protocol without by-products.

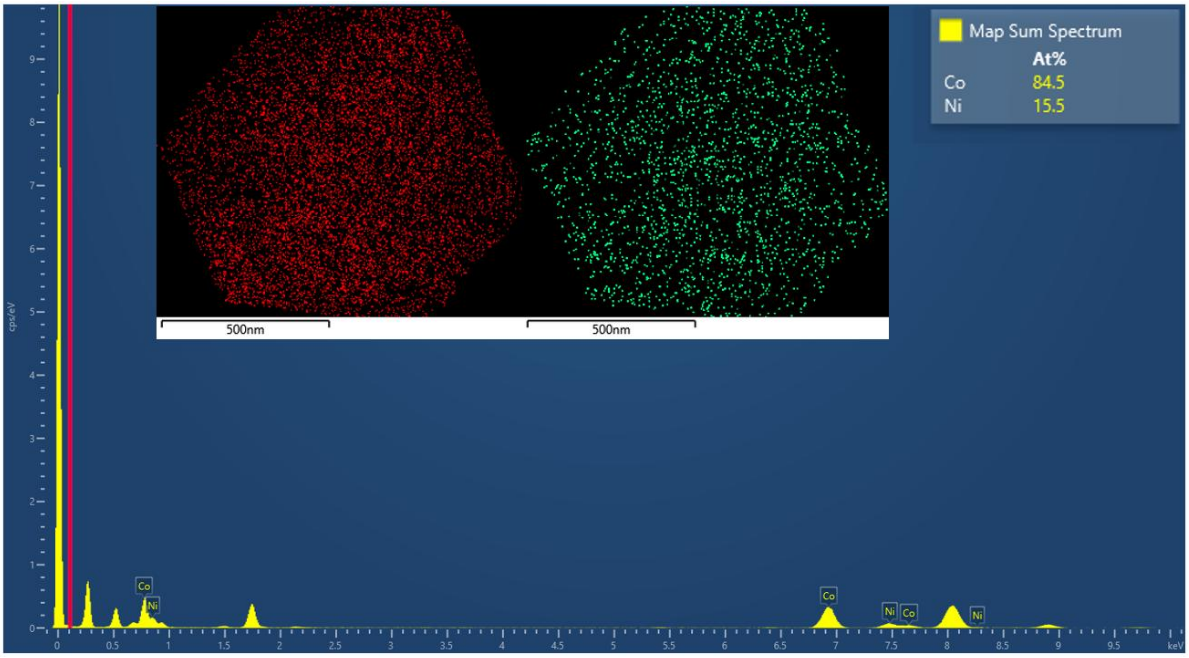

Figure S7: STEM-EDX spectrum and elemental Co and Ni mapping of $Co_{0.85}Ni_{0.15}$. The cobalt signal is shown in red, the nickel signal in green.

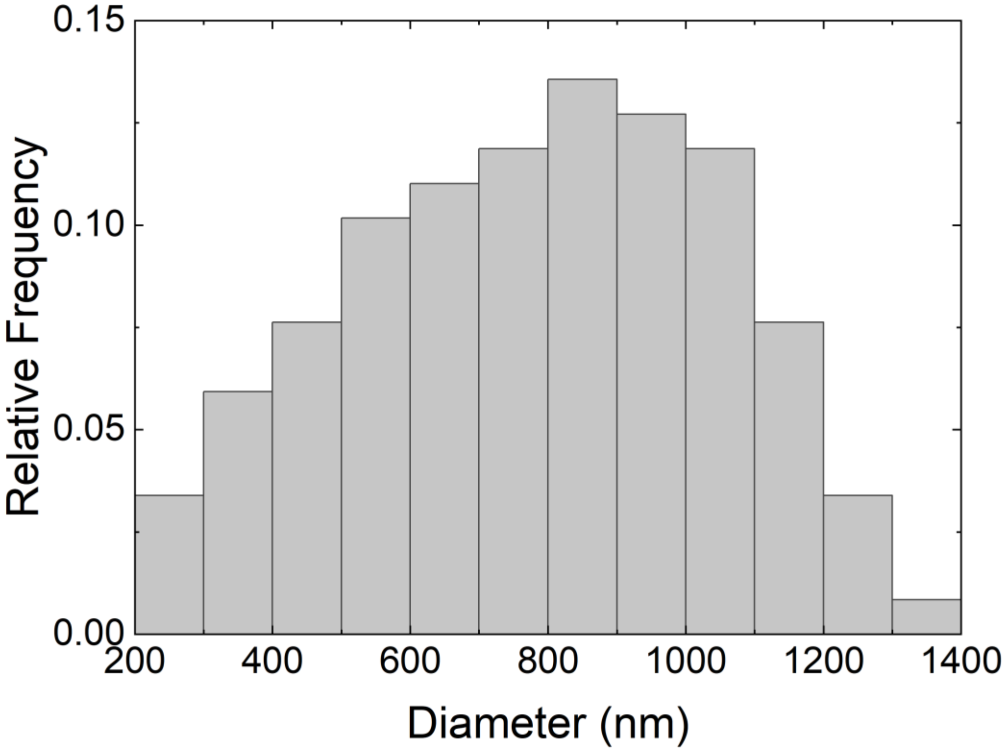

Figure S8: Size distribution of the $Co_{0.85}Ni_{0.15}$ nanoplatelets after the reduction process. Overall, 119 platelets were measured.

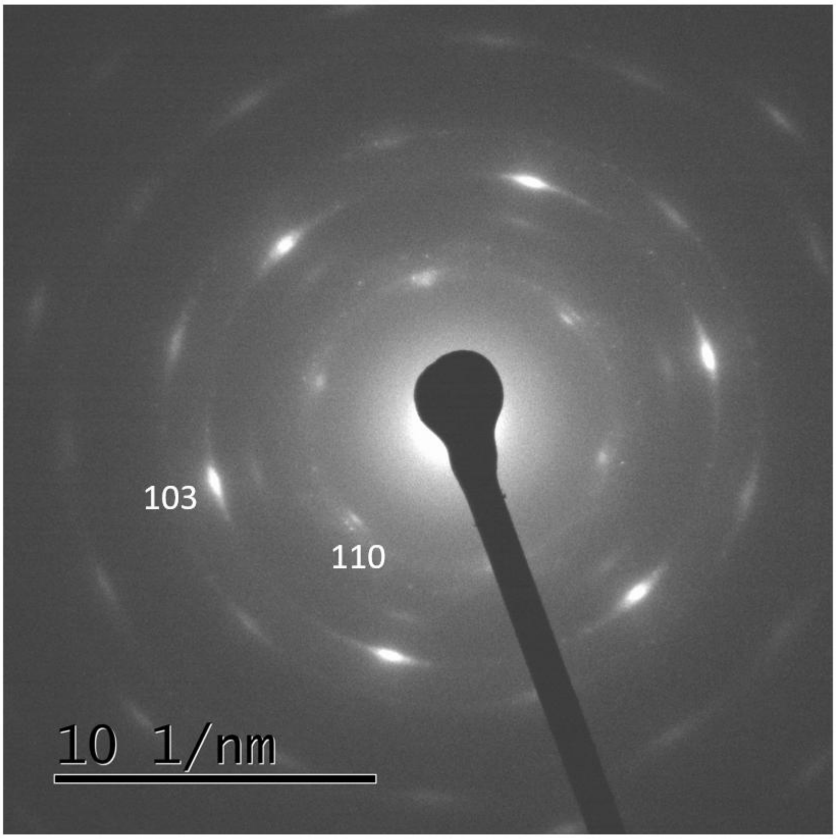

Figure S9: SAED of a magnetic nanoplatelet in the metallic state. The reflexes show a hexagonal lattice. The ring-like smearing of the reflexes indicate polycrystallinity with preferred orientations.

Table S1: Comparison of the calculated and measured lattice parameters.

Orientation	hcp $Co_{0.85}Ni_{0.15}$ (nm)	Measurement (nm)
110	0.2145	0.2146 ± 0.0019
103	0.1261	0.1261 ± 0.0017

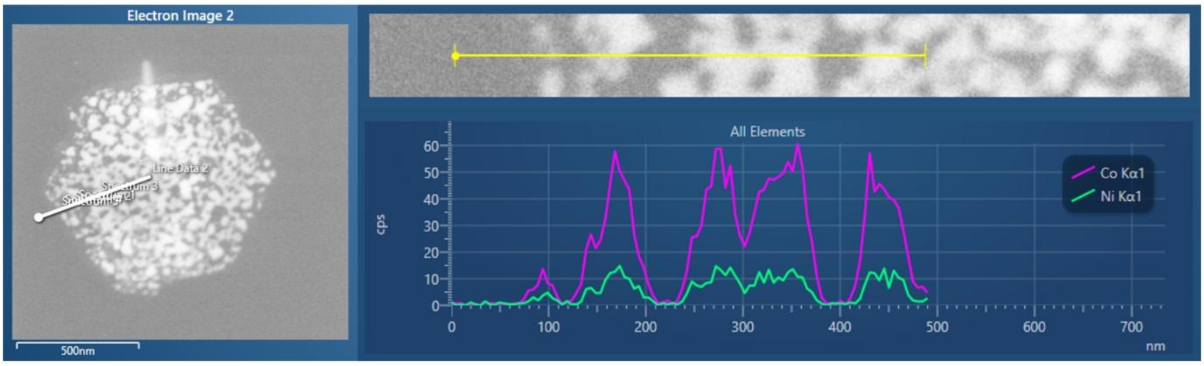

Figure S10: STEM image and EDX line scan of a $Co_{0.85}Ni_{0.15}$ defect platelet. The gaps are completely free of cobalt and nickel as indicated in the Co and Ni line scans. During reduction, this platelet on the TEM grid split into hundreds of irregular shaped nanoparticles maintaining the initial hexagonal shape.

Supporting Information Video File

The video file "LTEM-FocusSeries" attached to this article presents a $Co_{0.85}Ni_{0.15}$ platelet in Lorentz-TEM. The focus is adjusted between over- and under-focus conditions showing the Lorentz-contrast changes in the center and the rim of the platelet.

References

[1] L. Hu, Q. Peng, Y. Li, *Journal of the American Chemical Society* **2008**, *130*, 16136.

[2] Z. Liu, R. Ma, M. Osada, K. Takada, T. Sasaki, *Journal of the American Chemical Society* **2005**, *127*, 13869.

[3] J. Li, W. Zhao, F. Huang, A. Manivannan, N. Wu, *Nanoscale* **2011**, *3*, 5103.

[4] Z. Wang, M. S. Seehra, *Journal of physics. Condensed matter an Institute of Physics journal* **2017**, *29*, 225803.

[5] J. D. Rall, M. S. Seehra, *Journal of physics. Condensed matter an Institute of Physics journal* **2012**, *24*, 76002.

[6] C. D. Spencer, D. Schroeer, *Phys. Rev. B* **1974**, *9*, 3658.

[7] C. Tannous, J. Gieraltowski, in *Springer Handbook of Electronic and Photonic Materials* (Eds.: S. Kasap, P. Capper), Springer International Publishing. Cham **2017**, p. 1.
